# Ultra-small microorganisms in the polyextreme conditions of the Dallol volcano, Northern Afar, Ethiopia

**DOI:** 10.1038/s41598-019-44440-8

**Published:** 2019-05-27

**Authors:** Felipe Gómez, Barbara Cavalazzi, Nuria Rodríguez, Ricardo Amils, Gian Gabriele Ori, Karen Olsson-Francis, Cristina Escudero, Jose M. Martínez, Hagos Miruts

**Affiliations:** 1 Centro de Astrobiología (INTA-CSIC) Crtera. Ajalvir km 4 Torrejón de Ardoz, Madrid, 28850 Spain; 2 Dipartimento di Scienze Biologiche, Geologiche e Ambientali (BiGeA), Università di Bologna, Bologna, Italy; 3 Department of Geology, University of Johannesburg, Johannesburg, South Africa; 4 Centro de Biología Molecular “Severo Ochoa” Cantoblanco, Madrid, Spain; 5 IRSPS, Universitá d’Annunzio, Pescara, Italy; 6 Ibn Battuta Centre, Université Cadi Ayyad, Marrakech, Morocco; 7 School of Environment, Earth and Ecosystems Sciences, The Open University, Milton Keynes, UK; 8 Department of Earth Sciences, Mekelle University, Mekelle, Tigre Ethiopia

**Keywords:** Soil microbiology, Astrobiology, Soil microbiology, Astrobiology

## Abstract

The Dallol geothermal area in the northern part of the Danakil Depression (up to 124–155 meter below sea level) is deemed one of the most extreme environments on Earth. The area is notable for being part of the Afar Depression, an incipient seafloor-spreading center located at the triple junction, between Nubian, Somali and Arabian plates, and for hosting environments at the very edge of natural physical-chemical extremities. The northern part of the Danakil Depression is dominated by the Assale salt plain (an accumulation of marine evaporite deposits) and hosts the Dallol volcano. Here, the interaction between the evaporitic deposit and the volcanisms have created the unique Dallol hot springs, which are highly acidic (pH ~ 0) and saline (saturation) with maximum temperatures ranging between 90 and 109 °C. Here we report for the first time evidence of life existing with these hot springs using a combination of morphological and molecular analyses. Ultra-small structures are shown to be entombed within mineral deposits, which are identified as members of the Order Nanohaloarchaea. The results from this study suggest the microorganisms can survive, and potential live, within this extreme environment, which has implications for understanding the limits of habitability on Earth and on (early) Mars.

## Introduction

The study of environmental limits of life provides useful information for assessing the limits of habitability both on Earth and elsewhere in the Solar System^1,2^. Understanding, and defining these limits, using extreme terrestrial environments and Earth analogues sites^3,4^ is therefore a crucial step in selecting sites for future life detection missions.

**Figure 1:**
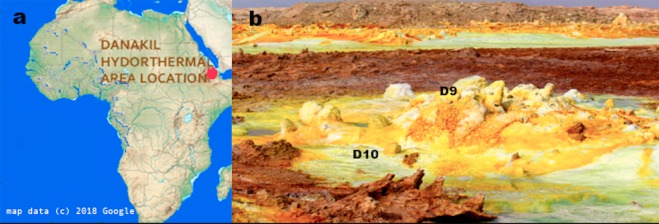
Study area. (**a**) Location map of the Dallol geothermal area (red dot) in the Danakil Depression (map data © 2018 Google). (**b**) Panoramic view of the sampling sites (D9: central small chimney and D10: water from the blue pool at the bottom of the chimney).

The Dallol geothermal area (14°14′21″N; 40°17′55″E) is located in the northern part of the Danakil Depression^5^. This is a narrow lowland salt plain (up to 124 m below sea level) running inland, quasi-parallel to the coast of the Red Sea (Fig. 1a), which formed when part of the Red Sea was isolated during the Pleistocene.

The area is located at the northern segment of the Afar triple junction and is characterized by an attenuated continental crust, which is less than 15 km thick with shallow (3–5 km deep) magma chambers beneath its axial zones^6,7^. The Dallol dome-shaped deposits formed as a result of the interaction between the evaporitic deposits and the shallow magma intrusions^7,8^.

The hydrothermalism of the Dallol area was possibly generated by a phreatic explosion in 1926. Since then volcanic episodes, seismicity and hydrothermal activity have been reported in the area^5^. As a consequence of geothermal activity^7,9^ several minerals, for example, pyrolusite, chlorargyrite, wurtzite and halite, precipitate forming colourful chimneys (Fig. 1b). It has been postulated that microbial activity does not play a major role in mineral precipitation and geochemical cycling within Dallol^10^.

At the surface, the water temperature at the source is above 100 °C and highly acidic (pH ~ 0). The resultant hot pools vary in color depending on the high metal concentration- (e.g., iron 35.6 g/L, copper 93 mg/l) (Table 1). A combination of these extreme chemical and physical parameters (e.g. temperature, pH, salinity and heavy metals) has resulted in a unique multi-extreme environment.

**Table 1:** Physico-chemical parameters for two sampling sites, D9 and D10 (concentrations in mg/l, average of three measurements). The units are stated as followed T: °C, Eh: mV; conductivity: mS/cm^2^.

Sample	D9	D10
pH	0.25	2.42
T	86	47
Cond	188	262
Eh	411	412
H_2_%	0.02	0.13
CO_2_%	0	0.15
Na	119333	89323
Mg	3408	5998
Al	383	533
P	10.1	14.9
K	8248	16594
Ca	3810	5927
Mn	538	950
Fe	19159	35652
Cu	40.6	93
Zn	39.6	72
Rb	11.9	20.9
Sr	71.1	116.7
Pb	1.4	0.2

First reported thermoacidophilic prokaryote was isolated from coal refuse piles^11^ and the first eukaryote from upper oxygenic zones of acidic geothermally heated waters in solfataras^12^. In 1995 two species of a moderately thermophilic archaea living around pH 0 were reported^13^.

Here, we describe for the first time morphological and molecular evidence of thermohaloacidophilic nanomicroorganism existing in this novel multi-extreme environment.

Due to the unique geochemistry and volcanic activity of Dallol^6,7^ it is an ideal analogue site for studying Martian hydrothermal environments, such as Nili Patera caldera^9^, where hydrothermal sinter deposits are found in direct association with volcanic activity^14^, and Gusev crater^15^, the landing site of the Spirit MER rover.

Despite the Dallol hot springs being deemed one of the most extreme environments on Earth the possibility of microbial life existing within the springs has not been studied.

## Results and Discussion

**Figure 2:**
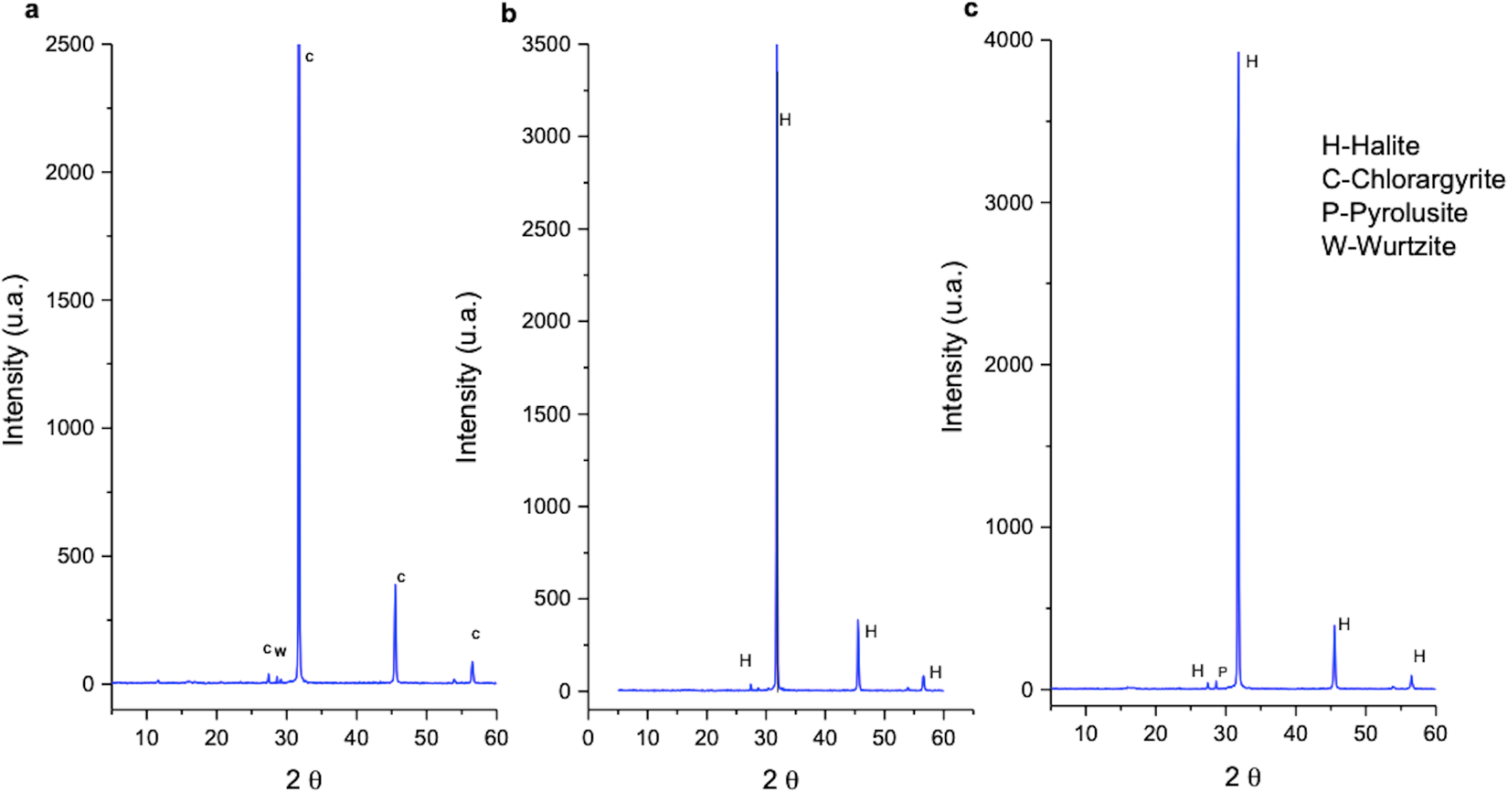
X-Ray Diffraction analyses showing sample D9 mineral composition. (**a**) chlorargyrite (**C**) and wurtzite (W); (**b**) halite (H), and (**c**) pyrolusite (P) and halite (H).

In this system many protuberances can be observed (Figs 1b and 6A–C), which are generated by the precipitation of minerals from the superheated underground solutions. The chimney selected for this study was Site D9 (Figs 1b and 6A) and composed of chlorargyrite (Fig. 2a), wurtzite (Fig. 2a), halite (Fig. 2b) and pyrolusite (Fig. 2c). Table 1 reports the physico-chemical parameters of Site D9 and the surrounding pool (Site D10) as shown in Fig. 6A. The pH and temperature between the two sites varied, for example at Site D9, the source of the hydrothermal fluid, the fluid was pH 0.25 and 86 °C compared to 2.42 and 47 °C at site D10.

**Figure 3:**
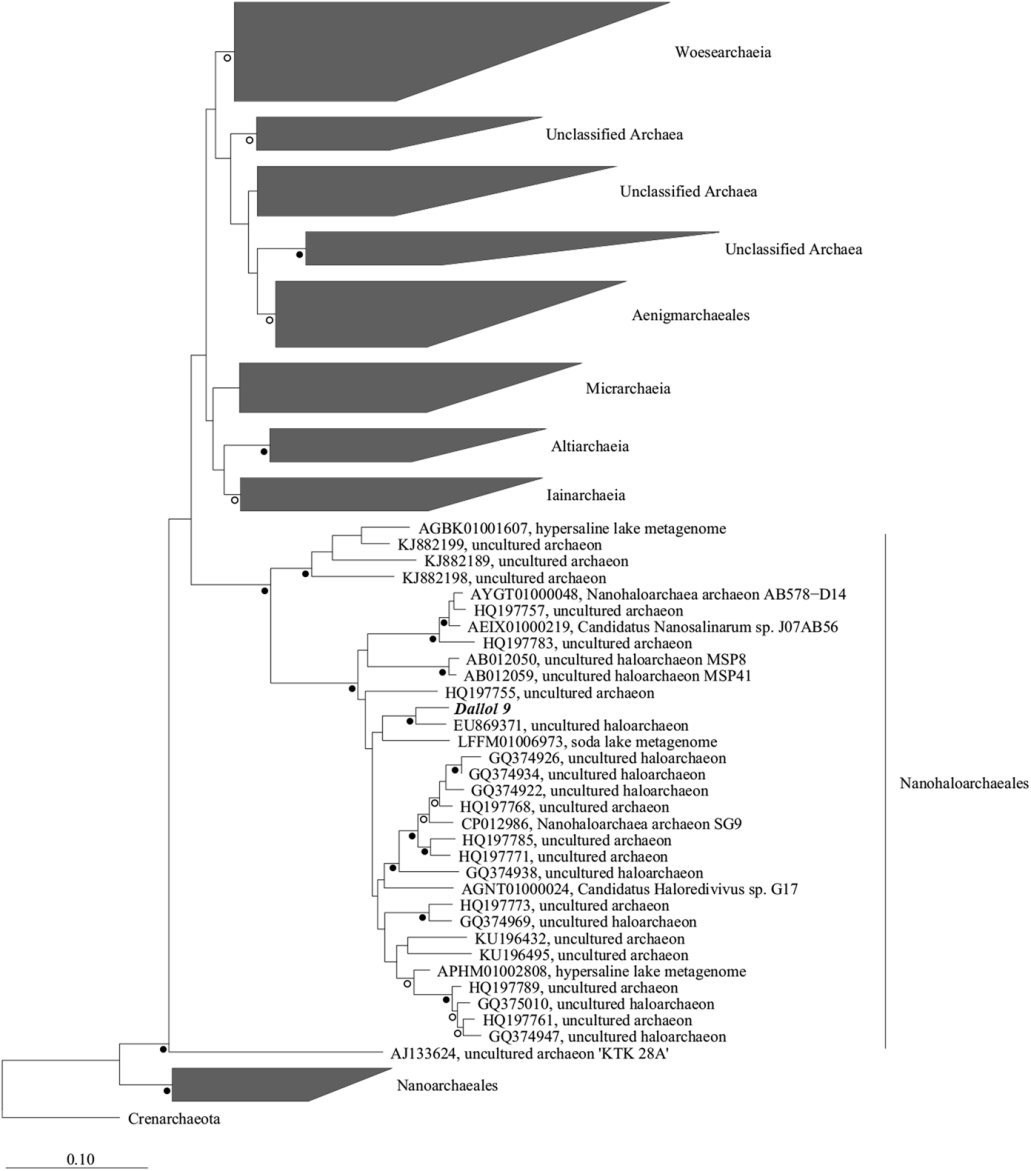
Neighbor-joining 16S rRNA phylogenetic tree of DPANN archaea. Open circles indicate bootstrap values between 70–89. Solid circles indicate bootstrap values bootstrap values higher than 90.

**Figure 4:**
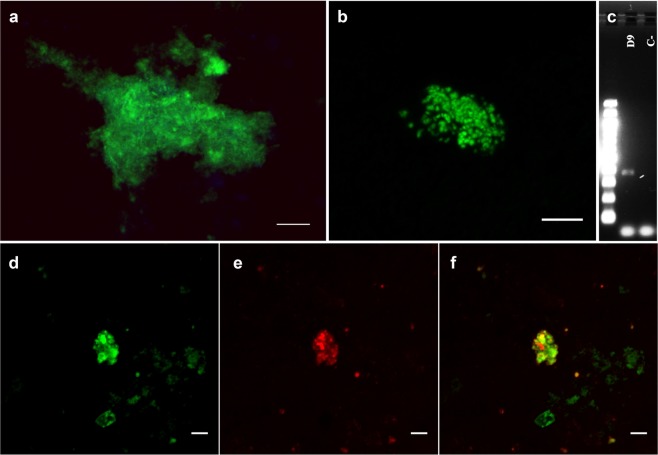
Living microorganisms at the Dallol hydrothermal outcrop. (**a**) SybrGold DNA staining of microorganisms retained in 0.2 µm filter before ultrasonication. (**b**) SybrGold DNA stain of microorganisms retained in 0.1 µm filter after ultrasonication. (**c**) Electrophoresis of archaeal amplified DNA from sample D9. (**d**–**f**) FISH images of Nanohaloarchaea microorganisms. In green, SybrGold DNA staining (**d**). In red, Narc1214 FISH probe signal (**e**). (**f**) d and e merge. Scale bars, 5 μm.

DNA was extracted from the salt precipitates at the hydrothermal fluid source (D9), 16S rDNA was amplified using Archaea primers (Fig. 4c) and phylogenetic analysis was performed. The OTU named Dallol 9 showed close similarity (95.8%) with the Nanohaloarchaea environmental clone ARDARCSS13 (GenBank accession number EU869371) (Fig. 3) which closely matched to uncultured sequences from Nanohaloarchaeales Order.

The clone ARDARCSS13 and Dallol 9 sequences are close to *Candidatus Holaredivivus* sp. G17 (Fig. 3) that has been described^16^ in a Bras of Port saltern pond (Alicante, Spain) as a photoheterotrophic microbe that present rhodopsin and photolyase. *Candidatus Holaredivivus* sp. G17 is capable to degrade polysaccharides, likewise presents typical genes of archaea. In relation to survival strategies in hypersaline environments, Haloredivivus sp. G17 is a salt-in strategist as the isoelectric point of his proteins shows^16^.

To confirm the presence of members of the Nanohaloarchaea group in the samples Fluorescence *In Situ* Hybridization (FISH) was carried out. Samples fixed *in situ* for FISH were sequentially filtered through 0.45, 0.22 and 0.1 µm filters to select for small (<0.1 µm) microorganisms. Inspection of the DNA stained material retained in the 0.2 µm filters with SybrGold suggested the presence of ultra-small microorganisms forming compact dense colonies, including mucilaginous films (Fig. 4a). Ultrasonication was further used to disintegrate the colonies prior to sequentially filtration. The ultra-small microorganisms passed through the 0.22 µm filter, but were again retained on the 0.1 µm filters (Fig. 4b). Figure 4c shows 16S rDNA amplification using universal archaea primers.

As it is shown in Fig. 4d–f, members of the Nanohaloarchaea group were detected with the Narc1214 FISH probe in the salt precipitates at the hydrothermal fluid source from D9, which corroborates the phylogenetic data.

**Figure 5:**
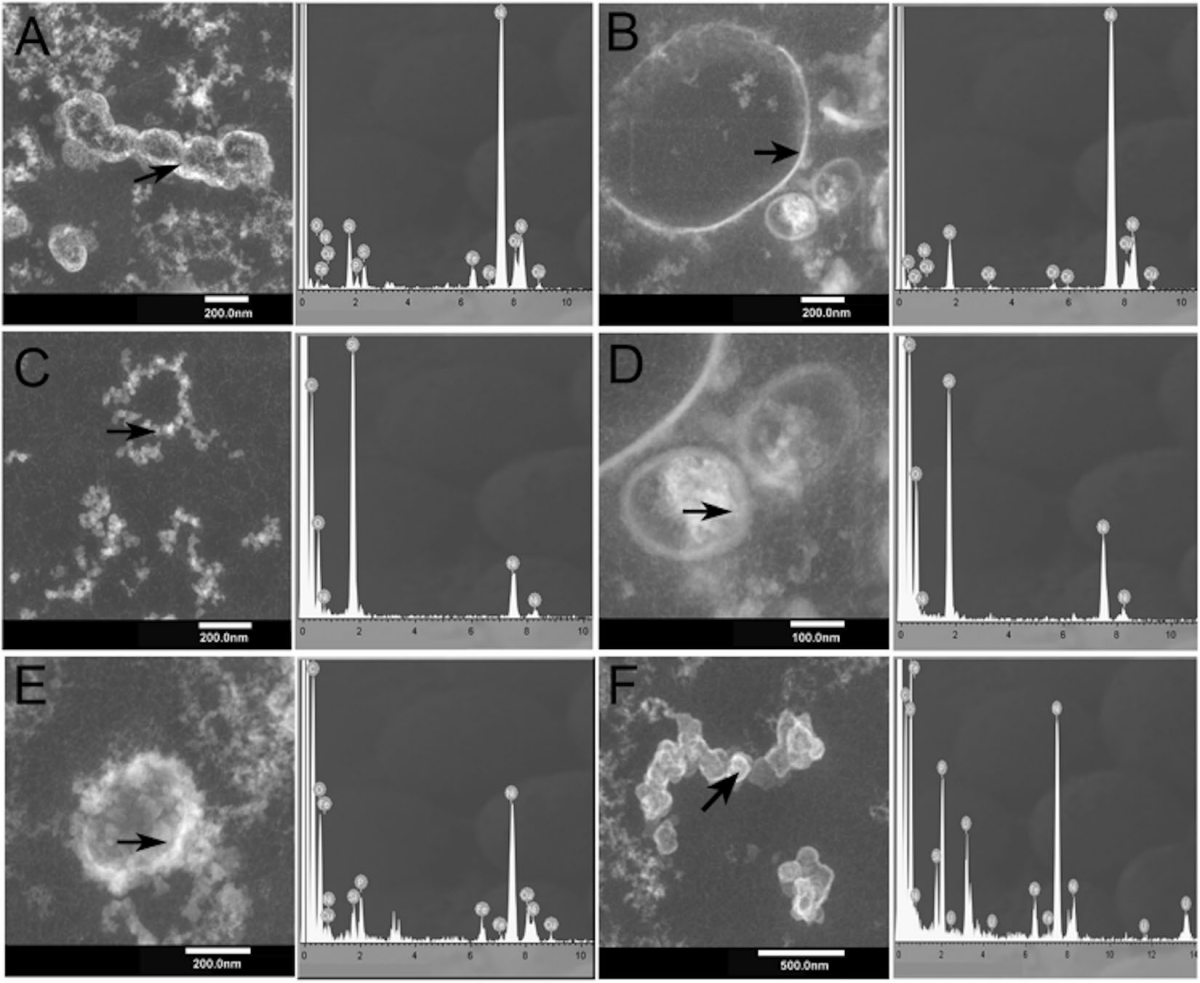
Transmission Electron Microscopy (TEM) images of sample D9 showing ultra-small biological morphologies. Arrows indicate where the EDX analyses were performed. Spectra show the high carbon content associated with high Si and Fe content in these spots.

**Figure 6:**
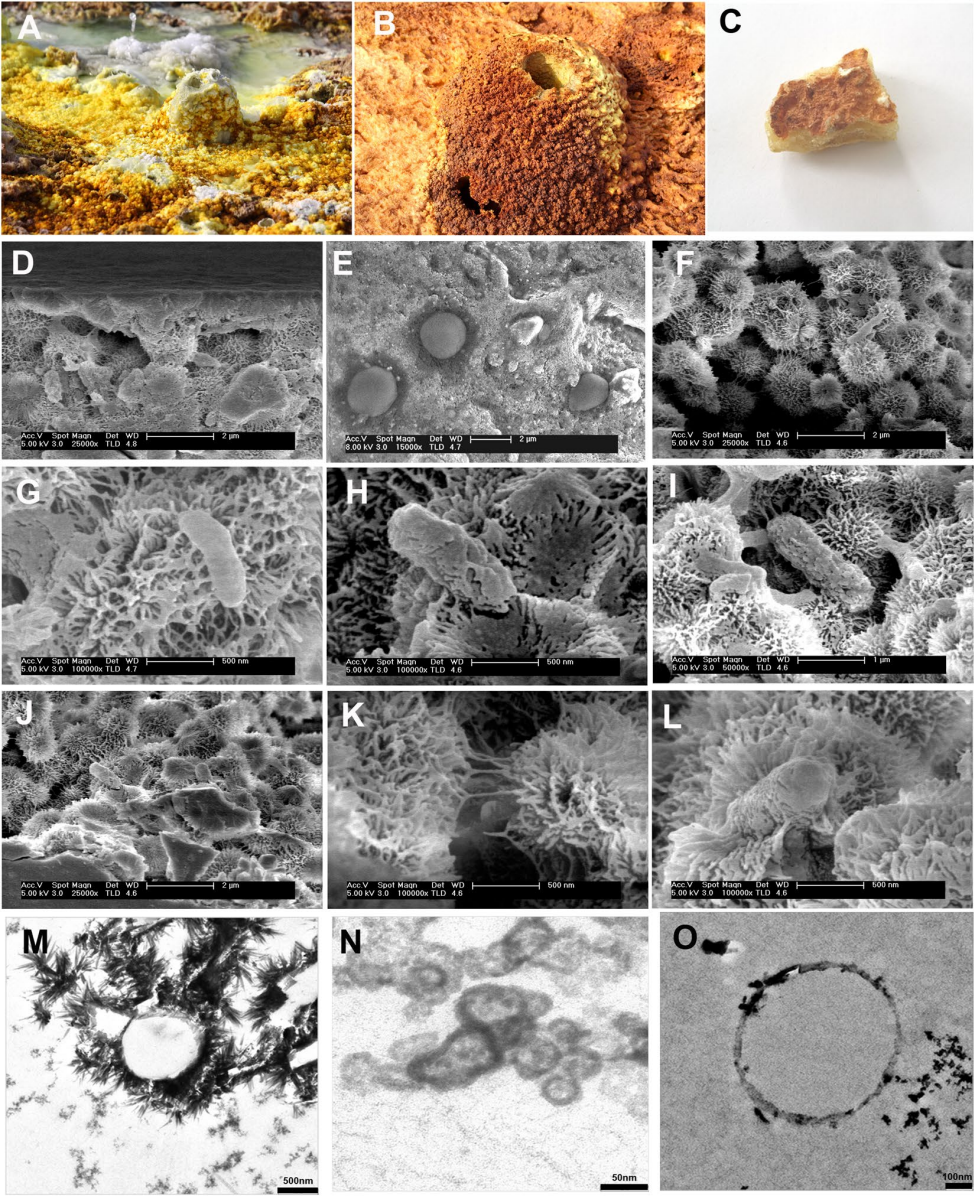
(**A**) General view of the sampling site, (**B**) the small chimneys (temperature of water 90 °C. (**C**) D9 sample from a small chimney in (**A**). (**D**–**L**) SEM and (**M**–**O**) Scanning TEM images of sample D9 showing the morphologies of ultra-small microorganisms entombed in the mineral layers.

The presence of ultra-small microorganisms was further investigated using Transmission Electron Microscopy (TEM) (Fig. 5), Scanning Electron Microscopy (SEM) and Scanning Transmission Electron Microscopy (STEM) (Fig. 6). Using these techniques, it was possible to identify ultra-small cellular morphologies (between 50 nm and 500 nm), which supports the molecular data (Figs 5 and 6). The Energy-dispersive X-ray (EDX) elemental microanalysis suggested that the cellular morphologies were unambiguously biological given their high carbon content (Fig. 5A–F). Whilst, SEM-EDX showed the presence of silica precipitates (Fig. 6G–M) surrounding and encrusting the ultra-small cellular morphologies (Fig. 5A–D), suggesting a hydrothermal origin with a high Fe content (Fig. 5E–F).

Figure 6 shows ultra-small microorganisms entombed within precipitated silica minerals (Fig. 6D–L). The cellular morphologies were associated with the needle-shaped crystals (Fig. 6M), which suggests a relationship between the microorganisms and the biomineralization process as has been previously reported in other acidic environments^17^.

The ultra-small microorganisms were entombed within the precipitated mineral structures (Fig. 6(D–F)). The mineral precipitation process gave rise to thin mineral layers (Fig. 6(D)). Figure 6(E,F) show an enlarged view of the thin mineral layers. Figure 6(G) shows ultra-small microorganisms, which were not covered by minerals, whilst Fig. 6(H,I) show ultra-small microorganisms partially covered with mineral precipitates. More biomineralized ultra-small microorganisms are visible in Fig. 6(J,K). Figure 6(M) shows STEM images with close views of ultra-small microorganisms surrounded by mineral needles and Fig. 6(F) shows mineral spheres resulting from the mineralization process. Needles with spike forms are observed in Fig. 6(J–L). Finally, Fig. 6(N,O) show ultra-small microorganisms surrounded by minerals in a close view using STEM.

## Concluding Remarks

We demonstrated the presence of living ultra-small microorganisms in a multi-extreme environment with adverse conditions for life: extreme low pH (0.25), temperature (90 °C), redox potential, salinity and heavy metals content. Molecular studies, electron microscopies observation and phylogenetic analyses of amplified rDNA sequences showed the presence of ultra-small microorganisms related to the Order Nanohaloarchaea. The ultra-small bacteria observed in the samples were morphologically spherical and entombed in the mineral layers that form the small chimneys in Dallol under high acidity and high temperature conditions. The saturation of salts and minerals in the superheated water, which results in precipitation and the formation of the chimneys may be influenced by entombed microorganisms. We described the presence of ultra-small microorganisms in a natural environment which is an Earth analogue of some regions of Mars such as Nili Patera Caldera^9,10^. The presence of life in the Dallol hot springs expands our understanding of the limits of habitability on Earth and beyond. However, future work is needed to understand how these nanobacteria survive in such an extreme environment and whether they play a role in geochemical cycling.

## Materials and Methods

### Sampling

The sampling campaign was carried out in January 2017 and samples were collected from a fumarole’s wall (14°14′21″N; 40°17′55″E) located at the main Dallol hydrothermal outcrop in the Danakil depression and a blue pool surrounding the small fumarole. Samples were collected aseptically, using sterilized spatulas and plastic aseptic materials in 12 mL sterile glass vials. The vials were completely filled to prevent head space. To ensure that the samples did not oxidize during transportation and storage, the vials were sealed with a septum tap, covered with parafilm tape and kept under anaerobic conditions. Samples were transported at room temperature.

### “*In Situ*” physico-chemical parameters measurement

Physico-chemical parameters (T: °C, Eh: mV; conductivity: mS/cm^2^) were measured “*in situ”* using a multi-parametric probe, YSI 556 MPS. Gas composition by gas chromatography and elemental composition using ICP-MS.

### Electron microscopy

Samples were fixed in 4% paraformaldehyde and 2% glutaraldehyde, in 0.1 M phosphate buffer (pH 7.2), for 2 h at room temperature. Fixed samples were washed three times in the same buffer and post-fixed with 1% O_s_O_4_ in water for 60 min at room temperature in the dark. After three washes in distillate water, samples were incubated with 2% aqueous uranil acetate for 1 h at room temperature, washed again, and dehydrated in increasing concentrations of ethanol 30, 50, and 70% 20 min each, 90% 2 × 20 min, and 100% 2 × 30 min at room temperature. Dehydration was completed with a mixture of ethanol/propylene oxide (1:1) for 10 min and pure propylene oxide 3 × 10 min. Infiltration of the resin was accomplished with propylene oxide/Epon (1:1) for 45 min and pure LR White resin (London Resin Company limited, England), overnight at room temperature. Polymerization of infiltrated samples was done at 60 °C for 2 days. Ultrathin sections of the samples were stained with uranyl acetate and lead citrate by standard procedures.

Four types of electronic microscopy techniques were used for this study: Scanning Electron Microscope (SEM) (JEOL-5600 coupled to an EDX, INCA) with an Energy Dispersive X-Ray Analyzer (EDX) and SEM-FI (Philips XL30-FEG); Scanning Electron Microscopy-Field Emission Gun (SEM-FEG) (Philips XL30-FEG); Transmission Electronic Microscope (TEM) (JEM-1010); TEM/STEM electronic microscope (JEOL 2100 K) with FEG. Samples were mounted on conductive graphite stubs and sputter and gold-coated in a Quorum, Q150T-S apparatus to ensure electrical conductivity and prevent charging under electron beams.

For SEM analysis, samples were also analyzed with EDX to obtain semiquantitative chemical data. A STEM unit was coupled to the microscope with acquisition of contrast images z (HAADF). The qualitative element composition of samples was determined using an INCA-X-SIGHT with a Si-Li detector (Oxford, England) with a detection limit of 10% for the main element. The operating energy was 200 kv. For TEM analyses, the instrument was operated at 200 kv (Catalysis Institute and PETROQUIMICA-CSIC), equipped with an energy dispersive X-RAY microanalysis instrument INCA (Oxford, England). The TEM/STEM was operated at 200 kv with an EDX, which was coupled to a STEM unit, with acquisition of contrast images z (HAADF). The same grids were used for both TEM and TEM-STEM.

### Fluorescence microscopy

Samples were fixed in 4% formaldehyde in phosphate-buffered saline (PBS) for 2 h and stored at 4 °C until further processing. Fixed samples were dispersed by 3 cycles of sonication of 30 sec, with a 30 sec break in between with 1 pulse per second (intensity of 20%). The samples were filtered sequentially through a 0.4 μm pore size polycarbonate filters, a 0.2 μm pore size filter and a 0.1 μm pore size filter (Millipore, Germany). Filters were pretreatment as previously described^18^. FISH was performed as described by Glöckner, *et al*.^19^ using Cy3 single-labeled Narc1214 probe^20^ (Biomers, Ulm, Germany). Stringencies were regulated adjusting formamide and NaCl concentration in hybridization and washing buffer at 30% (vol/vol) and 0.112 M respectively. Filters were counterstaining by incubation with SYBR® Gold (Molecular Probes, Eugene, OR, USA) 1X diluted in milliQ water for 15 min, before being mounted on glass a slide using Vectashield (Vector Laboratories, Burlingame, CA, USA): Citifluor (Citifluor, London, United Kingdom) (1:4). Samples were imaged using a confocal laser scanning microscope LSM710 (Carl Zeiss, Jena, Germany) equipped with diodo (405 nm), argon (458/488/514 nm) and helium and neon (543 and 633 nm) lasers. Fiji software^21^ was used to process images.

### DNA extraction and amplification of ribosomal genes

Samples were filtered through polycarbonate membranes (0.45, 0.2 and 0.1 µm diameter; Millipore, USA). Total genomic DNA was extracted from those membranes using the DNeasy PowerSoil Kit (Qiagen, Germany) as described in the manufacture’s instructions, and concentrated using a SpeedVac Concentrator. The quantity of the extracted DNA was analysed by fluorimetry using a Qubit 2.0 fluorometer (Thermo Fhiser Scientific, USA). Total genomic DNA were stored at −20 °C for sequencing.

Ribosomal genes were amplified using a primers set specific to amplify V2-V3 region of 16S rRNA in Archaea domain (Arch1F 5′-CGGRAAACTGGGGATAAT-3′ and Arch1R 5′-TRTTACCGCGGCGGCTGBCA-3′). PCR reactions were performed as described^22,23^. Gel electrophoreses (1% agarose Conda, Spain in 0.5X TBE buffer, 90 mV during 30 min and staining with GreenSafe Premium NZYTech) were carried out to check the size and quality of PCR products.

### Ribosomal genes library preparation and sequencing

Library preparation and 2 × 300 pair-end sequencing by Illumina MiSeq were made by Genomic Unit in Parque Científico de Madrid Foundation/FPCM (Madrid, Spain).

Total genomic DNAs was quantified by Picogreen. Then, an input of 16 pg of DNA and 27 cycles were used in a first PCR with Q5® Hot Start High-Fidelity DNA Polymerase (New England Biolabs, USA) in the presence of 100 nM primers for 16S amplification (5′-ACACTGACGACATGGTTCTACACCTACGGGNGGCWGCAG-3′ and 5′- TACGGTAGCAGAGACTTGGTCTGACTACHVGGGTATCTAATCC-3′, these primers amplify the V3-V4 region of 16S), 200 nM primers for Archaea amplification (5′-ACACTGACGACATGGTTCTACACGGRAAACTGGGGATAAT-3′ and 5′-TACGGTAGCAGAGACTTGGTCTTRTTACCGCGGCGGCTGBCA-3′),

After the first PCR, a second PCR of 15 cycles was perfomed with Q5® Hot Start High-Fidelity DNA Polymerase (New England Biolabs, USA) in the presence of 400 nM of primers (5′-AATGATACGGCGACCACCGAGATCTACACTGACGACATGGTTCTACA-3′ and 5′-CAAGCAGAAGACGGCATACGAGAT-[10 nucleotides barcode]-TACGGTAGCAGAGACTTGGTCT-3′) of the Access Array Barcode Library for Illumina Sequencers (Fluidigm, USA).

The amplicons were validated and quantified by a Bioanalyzer. An equimolecular pool was purified by gen extraction and titrated by quantitative PCR using the “Kapa-SYBR FAST qPCR kit for LightCycler480” with a reference standard for quantification. The pooled amplicons were denatured and added to the flowcell at a density of 9 pM. Clusters formed, which were sequenced using a “MiSeq Reagent Kit v3”, in a 2 × 300 pair-end sequencing run on an Illumina MiSeq sequencer.

### Detection of DPANN OTUs by microbiome analyse

Quality of reads was evaluated by means of FastQC software. PANDAseq Assembler was using for assembling forward and reverse reads and convert in a fasta file^24^. Sequencing data were processed using Qiime software package version 1.9.0^25^. High quality contigs were clustered into OTUs based on 94% sequence similarity with UCLUST. The first sequence for each OTU as the representative OTU, which were aligned using PYNAST^26^. The taxonomic identity of each phylotype was determined using the SILVA_132_QIIME database (https://www.arb-silva.de/download/archive/qiime/)^27^. Since Ultra small microorganisms are the target of this study, their representative sequences were filtered from biom table in order to analysis only these taxa using *filter_taxa_from_otu_table.py* and *filter_fasta.py* scripts in Qiime. Finally, the ARB software package^28^ was used to reconstruct the phylogenetic tree. The neighbour-joining with Felsenstein correction included in the ARB package was used for phylogenetic inference. The robustness of the reconstructed trees was evaluated by bootstrap analysis of 1000 resampled datasets.

## References

[1] Horneck G (2016). AstRoMap European Astrobiology Roadmap. Astrobiology..

[2] Stetter KO (2003). Extremophiles and their adaptation to hot environments. FEBS Letters..

[3] Fairén AG (2010). Astrobiology through the Ages of Mars: The Study of Terrestrial Analogues to Understand the Habitability of Mars. Astrobiology..

[4] Rothschild LJ (1990). Earth analogs for Martian life. Microbes in evaporites, a new model system for life on Mars. Icarus..

[5] Miruts H, Bheemalingeswara K, Jemal A (2016). A preliminary geological and generalized stratigraphy of western margin of northerm Afar depression, Dallol area, northern Ethiopia. Momona Ethiopian Journal of Science..

[6] Pagli C (2012). Shallow axial magmachamber at the slow spreading Erta Ale Ridge. Nat Geosci.

[7] Nobile A (2012). Dyke-fault interaction during the 2004 Dallol intrusion at the northern edge of the Erta AleRidge (Afar, Ethiopia). Geophys Res Lett.

[8] Cavalazzi B., Barbieri R., Gómez F., Capaccioni B., Olsson-Francis K., Pondrelli M., Rossi A.P., Hickman-Lewis K., Agangi A., Gasparotto G., Glamoclija M., Ori G.G., Rodriguez N., Hagos M. (2019). The Dallol Geothermal Area, Northern Afar (Ethiopia)—An Exceptional Planetary Field Analog on Earth. Astrobiology.

[9] Fawdon P (2015). The geological history of Nili Patera, Mars. JGR..

[10] Kotopoulou E (2019). A Polyextreme hydrothermal system controlled by iron: the case of Dallol at the Afar triangle. ACS Earth Space Chem..

[11] Darland G, Brock TD, Samsonoff W, Conti SF (1970). A thermophilic acidophilic Mycoplasma isolated from a coal refuse pile. Science.

[12] Brock Thomas D. (1978). Thermophilic Microorganisms and Life at High Temperatures.

[13] Schleper C, Pühler G, Kühlmorgen B, Zillig W (1995). Life at extremely low pH. Nature.

[14] Skok JR, Mustard JF, Ehlmann BL, Milliken RE, Murchie SL (2010). Silica deposits in the Nili Patera caldera on the Syrtis Major volcanic complex on Mars. Nature Geoscience..

[15] Ruff SW, Farmer JD (2016). Silica deposits on Mars with features resembling hot spring biosignatures at El Tatio in Chile. Nature communications..

[16] Ghai R (2011). New abundant microbial groups in aquatic hypersaline environments. Scientific reports.

[17] Oggerin M, Tornos F, Rodriguez N, Pascual L, Amils R (2016). Fungal Iron biomineralization in Río Tinto. Minerals.

[18] Ishii K, Mussmann M, Macbregor BJ, Amann R (2004). An improved fluorescence *in situ* hybridization protocol for the identification of bacteria and archaea in marine sediments. FEMS Microbiol Ecol..

[19] Glöckner FO (1996). An *In Situ* Hybridization Protocol for Detection and Identification of Planktonic Bacteria. Systematic and Applied Microbiology.

[20] Narasingarao P (2012). De novo metagenomic assembly reveals abundant novel major lineage of Archaea in hypersaline microbial communities. The ISME journal.

[21] Schindelin J (2012). Fiji: an open-source platform for biological-image analysis. Nat Methods..

[22] Cruaud P (2014). Influence of DNA extraction method, 16S rRNA targeted hypervariable regions, and sample origin on microbial diversity detected by 454 pyrosequencing in marine chemosynthetic ecosystems. Applied and Environmental Microbiology..

[23] Klindworth A (2013). Evaluation of general 16S ribosomal RNA gene PCR primers for classical and next-generation sequencing-based diversity studies. Nucleic acids research..

[24] Bartram AK, Lynch MD, Stearns JC, Moreno-Hagelsieb G, Neufeld JD (2011). Generation of multimillion-sequence 16S rRNA gene libraries from complex microbial communities by assembling paired-end Illumina reads. Applied and environmental microbiology..

[25] Caporaso JG (2010). QIIME allows analysis of high-throughput community sequencing data. Nature methods..

[26] Caporaso JG (2010). PyNAST: a flexible tool for aligning sequences to a template alignment. Bioinformatics..

[27] Quast C (2012). The SILVA ribosomal RNA gene database project: improved data processing and web-based tools. Nucleic acids research..

[28] Ludwig W (2004). ARB: a software environment for sequence data. Nucleic acids research.

