# Author Correction: Ultra-small microorganisms in the polyextreme conditions of the Dallol volcano, Northern Afar, Ethiopia

**DOI:** 10.1038/s41598-020-60960-0

**Published:** 2020-02-26

**Authors:** Felipe Gómez, Barbara Cavalazzi, Nuria Rodríguez, Ricardo Amils, Gian Gabriele Ori, Karen Olsson-Francis, Cristina Escudero, Jose M. Martínez, Hagos Miruts

**Affiliations:** 1 Centro de Astrobiología (INTA-CSIC) Crtera. Ajalvir km 4 Torrejón de Ardoz, Madrid, 28850 Spain; 2 Dipartimento di Scienze Biologiche, Geologiche e Ambientali (BiGeA), Università di Bologna, Bologna, Italy; 3 Department of Geology, University of Johannesburg, Johannesburg, South Africa; 4 Centro de Biología Molecular “Severo Ochoa” Cantoblanco, Madrid, Spain; 5 IRSPS, Universitá d’Annunzio, Pescara, Italy; 6 Ibn Battuta Centre, Université Cadi Ayyad, Marrakech, Morocco; 7 School of Environment, Earth and Ecosystems Sciences, The Open University, Milton Keynes, UK; 8 Department of Earth Sciences, Mekelle University, Mekelle, Tigre Ethiopia

Correction to: *Scientific Reports* 10.1038/s41598-019-44440-8, published online 27 May 2019

This Article contains an error. A Data Availability section was originally not included – it should appear as below:

**Data Availability**

Raw data generated for this study is in GenBank under accession number LR596613.1 and Short Read Archive with accession numbers ERX3426322 and ERX3426323.

